# Dynamical systems in computational psychiatry: A toy-model to apprehend the dynamics of psychiatric symptoms

**DOI:** 10.3389/fpsyg.2023.1099257

**Published:** 2023-02-03

**Authors:** Christophe Gauld, Damien Depannemaecker

**Affiliations:** ^1^Department of Child Psychiatry, University Hospital Lyon, Lyon, France; ^2^Institut des Sciences Cognitives Marc Jeannerod, UMR 5229 CNRS, Université Claude Bernard Lyon 1, Lyon, France; ^3^Centre National de la Recherche Scientifique (CNRS), Institute of Neuroscience (NeuroPSI), Paris-Saclay University, Gif-sur-Yvette, France; ^4^Aix-Marseille University, INSERM, Institut de Neuroscience des Systèmes (INS), Marseille, France

**Keywords:** psychiatric disorders, computational psychiatry, dynamical systems, symptoms dynamics, theoretical psychiatry

## Abstract

**Introduction:**

These last years, scientific research focuses on the dynamical aspects of psychiatric disorders and their clinical significance. In this article, we proposed a theoretical framework formalized as a generic mathematical model capturing the heterogeneous individual evolutions of psychiatric symptoms. The first goal of this computational model based on differential equations is to illustrate the nonlinear dynamics of psychiatric symptoms. It offers an original approach to nonlinear dynamics to clinical psychiatrists.

**Methods:**

In this study, we propose a 3+1 dimensions model (*x, y, z* + *f*) reproducing the clinical observations encountered in clinical psychiatry with: a variable modeling environmental noise (*z*) on the patient's internal factors (*y*) with its temporal specificities (*f*) and symptomatology (*x*). This toy-model is able to integrate empirical or simulated data from the influence of perceived environmental over time, their potential importance on the internal and subjective patient-specific elements, and their interaction with the apparent intensity of symptoms.

**Results:**

Constrained by clinical observation of case formulations, the dynamics of psychiatric symptoms is studied through four main psychiatric conditions were modeled: i) a healthy situation, ii) a kind of psychiatric disorder evolving following an outbreak (i.e., schizophrenia spectrum), iii) a kind of psychiatric disorder evolving by kindling and bursts (e.g., bipolar and related disorders); iv) and a kind of psychiatric disorder evolving due to its high susceptibility to the environment (e.g., spersistent complex bereavement disorder). Moreover, we simulate the action of treatments on different psychiatric conditions.

**Discussion:**

We show that the challenges of dynamical systems allow to understand the interactions of psychiatric symptoms with environmental, descriptive, subjective or biological variables. Although this non-linear dynamical model has limitations (e.g., explanatory scope or discriminant validity), simulations provide at least five main interests for clinical psychiatry, such as a visualization of the potential different evolution of psychiatric disorders, formulation of clinical cases, information about attracting states and bifurcations, or the possibility of a nosological refinement of psychiatric models (e.g., staging and symptom network models).

## 1. Introduction

Contemporary psychiatric nosology is based on categorical and static taxonomic distinctions. Thanks to the International Classification of Diseases, Eleventh Edition (ICD-11) (CIM, 2019) and the Diagnostic and Statistical Manual of Mental Disorders, Fifth Edition (DSM-5) (Association, 2013), the operationalization of psychiatric disorders within descriptive and categorical nosographies has been one of the most important advances in contemporary psychiatry (or “psychopathology”) (Kendler, 2017). Partly related to these specificities, such classification systems have major limitations that substantially impede scientific and clinical progress (Krueger and Bezdjian, 2009; Hyman, 2010). In addition to the difficulty in “carving the nature at its joints,” i.e., clearly demarcated psychiatric disorder entities as discrete taxa (Kendler, 2016), a large part of these limitations stems from the conception of psychiatric disorders as categorical kinds, aka separate entities stable over time (Zachar, 2000; Haslam and Ernst, 2002). In this way, categorical psychiatric disorders as they are present in contemporary psychiatric nosography have at least three main limitations. First, they do not consider the temporal dynamics of psychiatric symptoms (Hitchcock et al., 2022). For instance, the category of schizophrenia provides an overview of the clinical picture of a patient, but fails to identify the evolution of his/her symptoms as events unfolded day after day and week after week. Secondly, categorical disorders cannot account for the intrinsic non-linearity of the evolution of disorders, as it is perceived in patients in clinical practice. There are indeed phenomenological transitions in the clinical state of a patient, sometimes slow and sometimes brutal, which are difficult to explain by a single psychiatric category (Nelson et al., 2021). Finally, these psychiatric categories cannot capture the intra-individual variability of the symptoms of a patient. For example, a series of studies have shown that there may be up to 1,030 unique symptom profiles of major depressive disorder (Fried and Nesse, 2015), or with any combination and classification, up to 636,120 ways to have a post-traumatic stress disorder (Galatzer-Levy and Bryant, 2013).

### 1.1. Psychiatry and dynamical systems

Psychiatric disorders are thus dynamical conditions (Demic and Cheng, 2014). They may be conceived as evolving entities, varying over time under the pressure of allostatic loads (i.e., accumulations of external factors over time), according to the evolution of symptoms (e.g., a delirium reinforcing the interpretative mechanisms) or according to subjective perceptions. A flow of current research theoretically aims to show that psychiatric disorders can be modeled dynamically (Kelso, 1995). Dynamical models aim to provide a precise outline accounting for the evolution of various psychiatric disorders or conditions over time, according to different types of temporal evolutions. For instance, Schizophrenia Spectrum (SS), Bipolar and related Disorders (BD), Major Depressive Disorders (MDD) evolve by outbreaks and oscillations. Attention Deficit/Hyperactivity Disorders (ADHD), Autism Spectrum Disorders (ASD) are considered as more continuous. Others, as Obsessive-Compulsive Disorders (OCD), normal grief or Persistent Complex Bereavement Disorder (PCBD) raise the question of an evolution influenced by various contextual factors (Association, 2013). However, despite a number of theoretical expectations on dynamical systems (Nelson et al., 2017), these theories have been under-applied in psychiatry.

### 1.2. Three kinds of theoretical clinical proposals

We have identified the existence of at least four main types of non-modeled clinical theoretical proposals in the psychiatric literature. The first one corresponds to the 3P model (Wright et al., 2019). The 3P model is defined as a model considering 3 factors: predisposing, precipitating and perpetuating (Spielman, 1986). Predisposing factors makes the system sensitive to a stimulus, and depends on the prior state of the system states. Precipitating factors initiate the dynamics of psychiatric disorders under the action of a trigger (named “kindling,” see below). The interaction between the first two factors (the predisposing and precipitating factors) is sometimes called a “stress-diathesis” model. Perpetuating factors keep the system burnished despite the absence of stimuli. The 3P model allows to understand the evolution of patients from the early stages of neurodevelopment, and to visualize their evolution over the life-course as a function of the influence of the three aforementioned factors. To our knowledge, however, the 3P model has never been mathematized.

The second kind of clinical theoretical proposal corresponds to the kindling model. This clinical formulation, from the field of epilepsy, explains the manifestations of a relatively short stage of a psychiatric disorder. At a during fleeting moments of susceptibility (e.g., from a few hours to a few weeks), a triggering factor would lead to expressing the manifestations of this disorder. This psychiatric disorder bursts by successive acute manifestations on a relatively short time scale (Adamec, 1990). When the system is above a certain threshold, the kindling formulation brings together two parameters: an increase in the frequency of cycles of bursting, and a triggering of these cycles more and more independently of environmental factors (i.e., reflecting a phenomenon of sensitization).

The third kind of clinical theoretical proposal corresponds to staging models. Staging models are defined as psychiatric models aimed at distinguishing subgroups evolving by (successive) stages (McGorry et al., 2014). However, this proposal remains based on a linear conception of psychiatric disorders (Nelson et al., 2017). We insist on these notions of linearity and non-linearity because we are going to propose a model which is by definition non-linear. Non-linearity is used to describe a situation where there is no direct relationship between an independent variable (e.g., “the depression”) and a dependent variable (e.g., “anhedonia”), i.e., where it cannot be drawn a “straight-line” between them.

Finally, the fourth kind of clinical theoretical proposal corresponds to the conception of psychiatric disorders through the prism of dynamical systems, as we are going to explain, develop and use in this work.

### 1.3. Case formulations

In this article, we propose to use dynamical systems in order to build a computational model to apprehend the dynamics of psychiatric symptoms. Three methodological anchors allow this development: case formulations, wealth of dynamic systems and desire for a manipulable model.

First, the main goal of the use of dynamical systems is to computationally validate empirical observations made by clinicians and researchers of the psychiatric field. This computational model is based on case formulations of different psychiatric disorders and conditions and aggregated symptoms. We define (stereotyped) case formulations as the psychiatric disorders and conditions of a given patient. These are typical cases of clinical observation. Case formulations aim to model the characteristics of specific individuals, e.g., “an individual with an autism spectrum disorder.” This computational model is thus designed in a contingent way to exemplify the phenotypes of psychiatric disorders. These stereotyped case formulations are those described in the textbook of clinical psychiatry and transmitted to any clinician in his/her elementary formation, and as he/she can then observe it in his/her daily practice (corresponding to irrefutable and prototypical cases of dynamical evolutions of psychiatric disorders). Such case formulations serve as tools that help organize complex and contradictory information about a person (Eells et al., 1998). Thus, case formulations help to describethe stereotypical description of the temporal evolution of psychiatric disorders, and phenomenologically reproduce the empirical or simulated dynamics of psychiatric disorders (e.g., the clinically observed relationships between the psychiatric variables), as described in the empirical and historical descriptions of clinicians and researchers.

Such dynamical model will allow to learn about non-linear phenomena and instability, major variations related to fluctuations in initial conditions, phenomena of resilience and fragility or the attainment of tipping points (transitions) and steady states, attractors and oscillations between multiple stability in response to internal conditions or external stressors. It also incorporates the elements mentioned in the three previous kinds of clinical modeling, e.g., predisposing, precipitating and perpetuating factors, consideration of different time frames, sensitization (kindling) and stages (staging models) of psychiatric disorders.

First, current computational models found in the literature integrate biological factors, can be predictive, are interested in different time scales, but rare are the models that allow the common integration of all these variables. Studies are restricted to parameters such as noise from the environment (e.g., psychosocial stress) (Huber et al., 2000) or monovariable approaches (unlike our 4 variables) (Demic and Cheng, 2014). Moreover, computational psychiatry models also rarely integrate symptoms. Secondly, a common joke in the field of computational psychiatry reports that the number of articles promoting the theoretical promises of the field has exceeded the number of its empirical articles. Complex dynamical systems theories have been used to metaphorically explore psychiatric disorders (e.g., depression which can be understood as a metaphorical “stuck state” of emotional processing) (King et al., 1983; Boldrini et al., 1998; Bystritsky et al., 2012; Hayes et al., 2015; Sulis, 2021). However, this metaphor has largely remained highly theoretical and has only marginally resulted in a manipulable model based on the dynamical system (Durstewitz et al., 2021).

### 1.4. Main goals

Based on these theoretical considerations, no computational model considering both symptomatology, internal factors, environment and temporality (i.e., the four variables of the model presented below) seems to exist in the scientific literature. We seek to go beyond theoretical contributions by proposing a variable modeling environmental noise (*z*) acting on the patient's internal elements (*y*) with its temporal specificities (*f*) and symptomatology (*x*).

Here, we propose such a dynamical model as a structure able to receive simulated or empirical data, reproducing the phenomenological dynamics of psychiatric disorders. The whole interest of such a model is precisely to be able to get away from the traditional diagnostic categories to apprehend a multitude of empirical or simulated symptoms in a transdiagnostic way. The “toolbox” constituted by this model can, for instance, integrate both anhedonia and low mood (major depressive disorder category) and acoustico-verbal hallucinations (schizophrenia category). We believe that this granularity at the scale of the symptom is particularly important for clinical psychiatry.

Moreover, because of its desire to be generalizable to different types of disorders and adaptable to different datasets, this toy-model differs from a certain number of other dynamic models of psychiatric disorders found in the scientific literature (Durstewitz et al., 2021).

We propose here a toy-model to study the dynamics of psychiatric symptoms, which reduces complexity by considering aggregates of non-linear relationships between a limited number of variables depending on time: environment, internal elements (e.g., subjective phenomenological experiences or biology) and symptoms. Such a model is based on equations intended to reproduce case formulations. It produces an abstract representation of patients. It is not built on empirical data collected in research. Such a computational model is thus called a toy-model. In a toy-model, abstract values correspond to qualitative behaviors empirically perceived in clinical practice. As we will discuss, such a toy-model could only serve to apprehend, understand, or support debates on the possible dynamics of psychiatric disorders. As its name indicates, a toy-model is deliberately used to explain and make practical a behavioral function, like a box containing balls, which is considered as a toy-model allowing to understand, in a simplified way, both the solar system and the interactions between atoms. As has already been proposed in the context of epilepsy (Depannemaecker et al., 2021) under the name of “*epileptor”* (Jirsa et al., 2014) (which accounts for electrophysiological brain activity), this dynamical modeling of psychiatric symptoms, internal elements and environment could thus be qualified as a “*psychiator.”*

## 2. Methods

### 2.1. Research methodology

We propose a model based on dynamical system for psychiatric disorders. This model is based on the theory of dynamical systems, which offers a framework for modeling the evolution of variables as a function of time. We used a structure, able to receive simulated data, reproducing the phenomenological dynamics of psychiatric disorders. In order to avoid starting from psychiatric categories, we isolated variables from case formulations of different psychiatric disorders and conditions and aggregates of symptoms.

The model captures the temporal evolution of a phenomenon using mathematical differential equations, which intrinsically integrate temporality. Differential equations allow to calculate next states given a current state, depending on time. In a first-order differential system, the state of a variable at a time t is calculated based on its variation with respect to time t-Δt. The rate of amplitude changes over time will determine the time scale. Each equation composing the system can vary according to its own time scale. This notion of time scales is an important consideration in psychiatric disorders because psychiatric acute events occur on a much shorter scale (minutes, hour, day, weeks) than the longer time scale of the development and consequences of a psychiatric disorder (months, years, decades). Thus, to model psychiatric disorders, a fast sub-system helps to switch from a basic (healthy or already pathologically latent) state to a state with high level of symptoms. A slower system is needed to drive the transition between these states. Therefore, a slow-fast system is proposed, including these different time scales, in which an external input drives the transition between states.

We seek to infer the dynamical relationships that exist between the variables producing a psychiatric phenomenon. Thus, we describe the expected temporal trajectory of the variables, by including different relevant psychiatric aspects into the equations to obtain the desired phenomenological characteristics. The parameters are identify in order that the simulation dynamics correspond to clinical observations. The modeling of psychiatric disorders could have been carried out in other ways. Our primary goal is to computationally match clinical observations.

### 2.2. General presentation of the model

**Figure 1:**
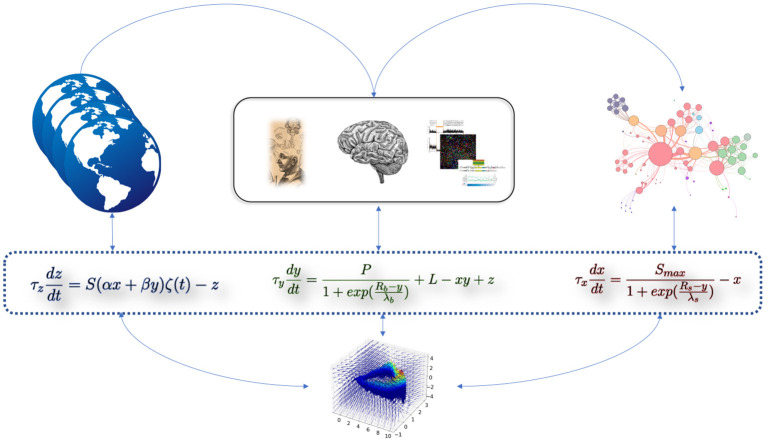
Cartoon representation of the computational dynamical toy-model of psychiatric disorders. The three equations are intrinsically linked by their interacting variables. The equation on the left (variable *z*) represents the effect of the external environment as perceived by the patient. The equation in the middle (variable *y*) represents the aggregation of internal elements (e.g., biological). The equation on the right (variable *x*) represents the intensity of symptoms. Empirical or simulated data can be “included” in these abstract variables (i.e., their precise identification does not change the model and its dynamics). The 3-dimensional phase space at the bottom of the figure corresponds to the toy-model presented in this study.

As schematized in Figure 1, the set of elements which should be integrated into the model, in order to account for the observed evolution of the psychiatric disorders modeled, should be: 1) A first variable *x*, which correspond to the intensity (or “apparent level”) of symptoms; 2) A second variable *y*, which aggregate the internal elements of a patient, interacting with the intensity of symptoms. This variable could refer to his/her “subjective state,” or “phenomenological state,” but also to biological elements (e.g., genetic data or brain morphological data). It is thus called a "potentiation variable,” because it potentializes the intensity of symptoms; 3) A third variable *z*, which correspond to the external environment as it is perceived and filtered by the patient. In addition to these variables, we add a fourth variable *f*, corresponding to the slow temporal fluctuations. This variable depends on *y* because the onset of symptoms depends primarily on the “subjective state" of the patient, i.e., its potentiation. In other words, there can be temporal fluctuations only if the patient describes subjective states, which are themselves at the origin of a potentiation of the symptomatology. In this computational model, we hypothesize that a slow accumulation in *f* allow a (slow) transition toward the pathological state. We seek to model the interactions between variables *x*, *y*, *z* (see Results for details of the effects of these interactions). The variables in the toy-model do not in any way prejudge any (top-down or bottom-up) causality, but rather are dependent variables interacting in a coupled way.

The computational toy-model is thus described by the following system of equations:


$$\begin{array}{l} \{\begin{array}{l} \tau_{x}\frac{dx}{dt}=\frac{S_{max}}{1+exp\left(\frac{R_{s}-y}{\lambda_{s}}\right)}-x\\ \tau_{y}\frac{dy}{dt}=\frac{P}{1+exp\left(\frac{R_{b}-y}{\lambda_{b}}\right)}+fL-xy-z\\ \tau_{z}\frac{dz}{dt}=S\left(\alpha x+\beta y\right)\zeta \left(t\right)-z\\ \tau_{f}\frac{df}{dt}=y-\lambda_{f}f \end{array} \end{array}$$

The literal descriptions of the parameters of the toy-model are given in Table 1.

**Table 1:** Description of the parameters of the toy-model.

**τ_*x, y, z, f*_**	**Time scales of the four equations**.
*S*_*max*_	Maximal level of symptoms.
*R*_*s*_ and *R*_*b*_	Sensitivity: the difficulty in triggering the system. Rs refers to sensitivity in terms of symptoms. Rb refers to sensitivity in terms of internal elements (or potentiation).
λ_*s*_ and λ_*b*_	Slope of the symptom and internal elements (or potentiation) curves. λ_*s*_ corresponds to the increase in the intensity of symptoms x as a function of the subjective state of the patient y. λ_*b*_ corresponds to the increase in the internal elements (or potentiation) y as a function of the environment z.
*P*	Maximal rate of internal elements of the systems: the aggregation of biological, psychological or subjective/phenomenological elements. It refers to the fabrication of the semantic configuration of a phenotype from a set of biological signals. It is an element of potentiation of symptoms.
*L*	Level of predisposing factors: it contributes to a permanent shift in the internal elements (potentiation).
λ_*f*_	Scaling factor of the slow evolution of the fluctuations affecting *L*
*S*	Overall sensitivity level to the environment.
α and β	Weight of the effect of the variable x and y on the perception of the environment

### 2.3. Equation 1: Modeling of symptom intensity

The first Equation (1) can be understood as: "The intensity of symptoms increases due to subjective state *y* of the patient, and saturate to a maximal value *S*_*max*_” (i.e., referring to a model with sigmoïdal function).


(1) $$\begin{array}{l} \tau_{x}\frac{dx}{dt}=\frac{S_{max}}{1+exp\left(\frac{R_{s}-y}{\lambda_{s}}\right)}-x \end{array}$$

If nothing participates to maintain high intensity of symptoms, the intensity of symptoms decreases over time (modeled with the exponential decay −*x*). The evolution of the intensity of symptoms occurs with the time scale of τ_*x*_. The *R*_*s*_ parameter corresponds to the sensitivity, i.e., the difficulty in triggering the system in terms of potentiation (i.e., if *R*_*s*_ is high, the appearance of symptoms occurs only for a very high value of the variable *y*). It can be seen as a form of sensitivity (or propensity) to develop symptoms depending on the internal elements. The λ_*s*_ parameter corresponds to the increase in the intensity of symptoms *x* as a function of the internal elements of the patient *y*, which is therefore almost linear in the middle of the curve (λ_*s*_ is the slope of the symptom curve where the sigmoïd is centered).

### 2.4. Equation 2: Modeling of internal elements


(2) $$\begin{array}{l} \tau_{y}\frac{dy}{dt}=\frac{P}{1+exp\left(\frac{R_{b}-y}{\lambda_{b}}\right)}+L-xy-z \end{array}$$

The second Equation (2) refers to the internal elements of a patient. The variable *y* evolves on the time scale τ_*y*_, and depends on the elements described below. The first term ($\frac{P}{1+exp\left(\frac{R_{b}-y}{\lambda_{b}}\right)}$) may be seen as the effect of the aggregate of internal elements underlying elements which have a dynamical effect depending on the state of the patient. A maximal fixed level of potentiation *P* corresponds to the subjective level of a patient allowing the existence of symptoms. In the Cambridge model, it could be seen as the “primordial soup,” i.e., the making of the semantic configuration of a phenotype from a set of biological signals (Berrios and Chen, 1993). In other words, *P* refers to the influence of the internal elements on the expression of symptoms through the variable *y*. The *R*_*b*_ and λ_*b*_ parameter are interpreted as for the intensity of symptoms but regarding internal elements (i.e., in terms of potentiation). The parameter *L* corresponds to the level of predisposing factors that contribute as a permanent shift in the potentiation. This parameter gives the baseline of predisposition for a psychiatric disorder. It corresponds to the basic level toward which the system tends when the intensity of symptoms diminished. The decay in time of this state potentiation being faster soon after paroxysmal symptomatic period, the decay is model by (−*xy*). Finally, the variable *y* is influenced by the perceived environment through *z*.

### 2.5. Equation 3: Modeling of perceived environment


(3) $$\begin{array}{l} \tau_{z}\frac{dz}{dt}=S\left(\alpha x+\beta y\right)\zeta \left(t\right)-z \end{array}$$

The third equation refers to the environment (or external world) perceived by a patient, modeled by the variable *z* (Equation 3), which evolves with a time constant τ_*z*_. It depends on the overal sensitivity level *S*, and the joint effects of symptoms *x* and the potentiation *y* respectively pondered by factor α and β. The factors α and β may be positive or negative depending on the type of psychiatric disease considered. The perceived environment integrates the equation as external noise ζ(*t*), set between –1 and 1 with Gaussian distribution. The release occurs with an exponential decay (−*z*).

### 2.6. Equation 4: Modeling of temporal specificities

A fourth equation can be added to model slower processes of psychiatric disorders. This equation is equivalent to a change of a parameter over time to capture elements on a much longer timescale, especially at the scale of a lifetime (Equation 4):


(4) $$\begin{array}{l} \tau_{f}\frac{df}{dt}=y-\lambda_{f}f \end{array}$$

This equation could be adapted according to the fluctuations of the values of λ_*f*_). These fluctuations can create oscillations, or slow evolutions of other variables over the long term. This is a variable of slowness, which interacts at a longer time with the other three variables evolving more rapidly. The variable depends on the internal potentiation *y*, and affect the latter as a multiplicative factor of *L*, the level of predisposing factors. Thus, the differential equation of *y* become (Equation 5):


(5) $$\begin{array}{l} \tau_{y}\frac{dy}{dt}=\frac{P}{1+exp\left(\frac{R_{b}-y}{\lambda_{b}}\right)}+fL-xy-z \end{array}$$

### 2.7. Set of constraints applied to the model

Due to the structure of these equations, we have to consider a set of constraints.

First, we are looking for a system representing several states, accounting for phase transitions: 1) of psychiatric states below a first threshold delimiting a state of health and a pathological state; 2) of psychiatric states above the threshold of psychiatric disorders; 3) of psychiatric states corresponding to the maximum intensity of symptoms, i.e., the most intense state of crisis describable for a disorder.

Secondly, configurations containing negative *x* (the intensity of symptoms) and *y* (the “subjective state” of the patient, a variable of potentiation) are not considered, as they are not (patho)physiologically plausible.

Thirdly, the rate of the noise ζ(*t*) is chosen at 0.01, meaning that the perceived environment variable *z* changes every 0.01 days (noise will be generated every 14.4 min). It is a compromise between the duration of variability of the symptoms of psychiatric disorders and their environment (i.e., considering a psychological state change every 14.4 min). In other words, the model provides a smoothness of 14.4 min, i.e., informs about potential changes in its variables approximately every quarter of an hour. This contingent choice captures most symptomatic variations of psychiatric disorders, but it does not record environmental noise without clinical value. For instance, a longer time (e.g., 12 h) would have missed potentially important information like mood variations during the day in the case of cyclothymia or behavioral disorders, while a shorter time (e.g., 10 s) would have captured too much noise without clinical value like emotional reactions to any life event.

Fourthly, the *S*_*max*_ parameter is fixed on a Likert scale (steps from 0 to 10). In the simulations, we saturate the scale to 10, to challenge the system to design maximum symptom intensity. Conversely, the other parameters cannot be quantified or bounded, because they depend on each patient specifically.

We use an Euler integration method with *dt* = 0.01 for the simulations given in the Results section.

### 2.8. Presentation of the simulations

In the following section, we will perform four simulations based on four case formulations to verify that the model captures the following stereotypical dynamics of psychiatric conditions: a healthy condition, a schizophrenia spectrum disorder, a rapidly cycling bipolar disorder, and a persistent complex bereavement disorder.

From the observed dynamics of simulations of these four case formulations of psychiatric disorders, conditions and aggregates of symptoms, and based on this set of equations, we will propose to identify contingent relative threshold values (maximum and minimum) for each of the 13 parameters of the *x, y, z* equations of model. These values will be identified empirically to be consistent with clinical observations. We will add for each of these simulations an external event that acts as an environmental trigger, not related to the patient. We consider a practical model which includes variations between its limit cycles and its fixed points, with an influence of the noise varying the characteristics of the system, and potentially several bifurcations.

Finally, in addition to these four simulations, we will propose a fifth simulation in which we visualize the effect of a therapeutic action according to knowledge and stereotyped case formulations.

## 3. Results

We used a toy-model built on differential equations to simulate the dynamics of psychiatric conditions and disorders through case formulations. Depending on the variability of the parameters handled in this toy-model, various dynamics of different psychiatric disorder could be modeled. If empirical data from research has been incorporated into the model, although each of these conditions tends to be as stereotyped as possible relative to empirical observations of clinical practice, each condition could be dynamically different according to the interindividual variations of the patients. Thus, in each of the following case formulations, based on observation of stereotypical cases, we can identify that each dynamic could be observed when the value of a parameter increases or decreases.

### 3.1. Identification parameter values for the simulations

**Figure 2:**
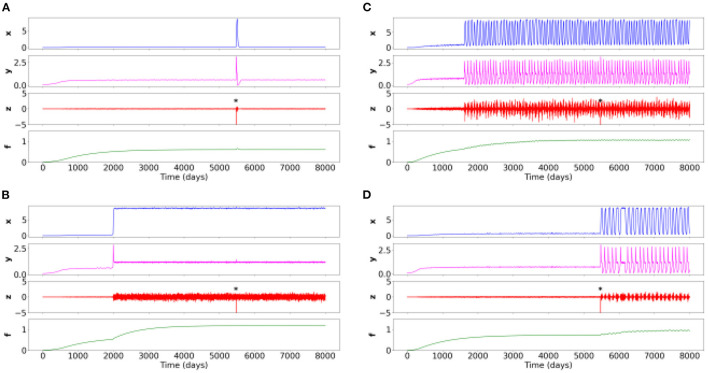
Time series of the four simulations for different sets of parameters. Negative events from the environment (variable *z*) are labeled with an asterisk. **(A)** Healthy situation: the negative event creates transient symptoms (*x*), potentiation of internal elements (*y*), and environmental sensitivity, and then returns to the baseline healthy level. **(B)** Constant symptom pathology (e.g., schizophrenia spectrum): symptoms appear at some point in life when potentiation has increased due to slowly changing accumulation variable *f*. The pathology is strongly expressed when the negative event occurs, and weakly affects the other variables. **(C)** Oscillating symptom pathology (rapid cycling in bipolar disorder): symptoms appear at some point in life when potentiation has increased due to a slow changing accumulation variable *f*. The disorder is strongly expressed when the negative event occurs which weakly affects the other variables. **(D)** A disorder based on oscillating symptoms (persistent complex grief disorder): a negative event triggers and stabilizes the oscillations despite the disappearance of this event.

Constrained by clinical observations, simulations of the four following psychiatric conditions (named here Figures 2A–D) provide relative parameter values which correspond to the maximum and minimum thresholds found empirically in order to obtain variable behavior in the simulations which are consistent with case formulations (Table 2).

**Table 2:** Values of the 13 parameters for the 4 simulations (or case formulations).

	***S*_*max*_**	***R*_*s*_**	**λ_*s*_**	**τ_*x*_**	***P***	***R*_*b*_**	**λ_*b*_**	***L***	**τ_*y*_**	***S***	**α**	**β**	**τ_*z*_**
Figure 2A	10	1	0.1	14	10	1.04	0.05	0.2	14	4	0.5	0.5	1
Figure 2B	10	1	0.1	14	10	0.904	0.05	0.2	14	4	0.5	0.5	1
Figure 2C	10	1	0.1	14	10	1.04	0.05	1.01	14	10	0.5	0.5	1
Figure 2D	10	1	0.1	14	10	1	0.05	0.6	14	4.5	0.5	0.5	1

For all simulations λ_*f*_ = 1 and τ_*f*_ = 720.

### 3.2. Dynamics of psychiatric disorders: Simulation results

#### 3.2.1. Case formulation 1: Healthy situation

In this case formulation, described in the panel (a) of the Figure 2, the modeled patient through the toy-model is in one possible healthy state. The corresponding parameters are given in Table 2, which thus provide the basic relative threshold values from which other psychiatric conditions will evolve.

At a random time of 5,500 days (about 15 years), a potentially destabilizing life event occurs. It could be, for instance, the death of a loved one. However, given the healthy characteristics of the modeled patient (depending on its different values of *x* and *y*), this event causes at most a normal grief, with a brief resolution of the symptoms. After the effect of the perturbation, all variables come back to their healthy initial level.

#### 3.2.2. Case formulation 2: Schizophrenia spectrum

In this case formulation, described in the panel (b) of the Figure 2, the patient would be diagnosed with a schizophrenia spectrum.

We identify that these dynamics could be observed when the *R*_*b*_ decreases, corresponding to a decrease in resistance. Thus, less resistance leads to an increase in the potentiation (*y*) and thus to a more intense development of symptoms (*x*).

At the random time of 5,500 days, in the absence of any intervention, a potentially destabilizing life event occurs. After this event, the symptoms persist due to different patient parameters simulated. We observe the complete absence of responsiveness to environmental stimuli.

#### 3.2.3. Case formulation 3: Rapid cycles in bipolar disorder

In this case formulation, described in the panel (c) of the Figure 2, the patient would be diagnosed with a rapidly cycling bipolar disorder.

Here, the *R*_*b*_ (i.e., sensitivity or resistance) is different from the formulation box (b), but identical to (a). However, the *P* (i.e., base level of potentiation) is much higher in this formulation case. This difference leads to the occurrence of rapid cycles. Moreover, the *S* (overall sensitivity level to the environment) is very high: the individual perceives his/her environment in a very senstive way.

Each complete cycle lasts approximately 100 days, with a symptom plateau lasting approximately 15 days, similar to what can be found in clinical practice. Finally, we retrieve that despite the presence of an intense life event, there is no change in the patient's sensitivity to his/her environment or in the intensity of the symptoms.

#### 3.2.4. Case formulation 4: Persistent complex bereavement disorder

In this case formulation, described in the panel (d) of the Figure 2, the patient would be diagnosed with a Persistent Complex Bereavement Disorder (PCBD).

In the PCBD, it is precisely the onset of an intense life event that causes this disorder. However, unlike the situation (a) in which the intensity of symptoms and the sensitivity to the environment returns to the threshold of normality over time, the patient modeled in this case formulation continues to have mood fluctuations.

This case formulation is thus close to the healthy situation, except for the *P* which is slightly higher, with a slightly lower resistance *R*_*b*_: this slight shift leads to the non-return to the healthy state.

#### 3.2.5. Action of psychiatric therapeutics on different psychiatric disorders

**Figure 3:**
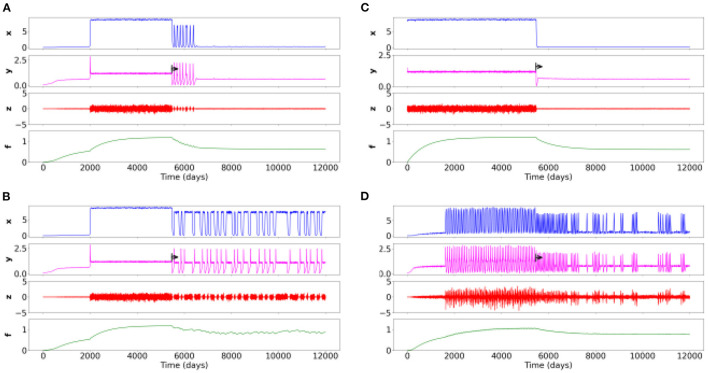
Time series of four simulations for different sets of parameters with a therapeutic action on different psychiatric disorders. The black arrow corresponds to the beginning of the treatment. **(A)** Schizophrenia spectrum with effective treatment. **(B)** Schizophrenia spectrum with insufficient treatment. **(C)** Childhood-onset disorder (e.g., neurodevelopmental disorder) treated with a fast-acting drug (e.g., methylphenidate) or another therapy. **(D)** Rapid cycling bipolar disorder with an ineffective treatment but with an action on the frequency, regularity and intensity of cycles.

In the Figures 3A, B, correspond to the Figure 2B, aka to the schizophrenia spectrum. In the Figure 3A, the given treatment is relatively efficient, but its effect is transient after some oscillations. This is the stereotypical case of antipsychotic treatments in schizophrenia, which take effect after several weeks and require adjustments related to early relapses.

In the Figure 3B, the treatment does not work well but enable brief moments of improvement and a slightly lower overall symptom intensity.

In the Figure 3C, the high intensity of symptoms present at the beginning of the period immediately decreases (on/off effect). This case formulation has a symptomatology which appears from birth, as is the case with, for instance, attention deficit disorders with or without hyperactivity. It could then be a treatment with (for instance) methylphenydate, having a rapid efficacy in this disorder/condition.

In the Figure 3D, corresponds to Figure 2C, i.e., rapid cycling bipolar disorder. We find with the treatment (e.g., lithium) an increase of healthy state over time. Note however that the treatment is insufficient, but it still changes the frequency, regularity and intensity of cycles.

## 4. Discussion

**Figure 4:**
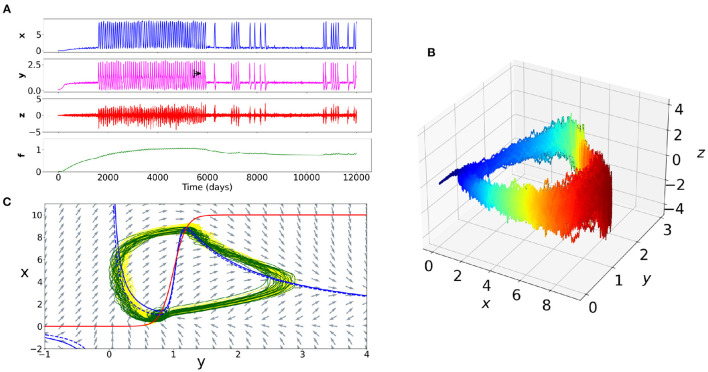
Example of the dynamics of a simulation of a rapid cycling bipolar disorder. **(A)** Time series of the four variables. The black arrow corresponds to a therapeutic action on the internal elements of the patient (e.g., on neurotransmitters). The intensity of the symptoms becomes thus less frequent. **(B)** Trajectory in the phase space of the variables *x, y, z*. The blue color corresponds to a decrease in symptoms and the red color to an increase in symptoms. The cyclic nature of the trajectory appears clearly. The path at the onset of the peak of symptoms intensity is different than at the offset. **(C)** Plane of a subspace of the system. The NullClines of the equations *x* and *y* appear respectively in red and blue colors. The blue dotted line corresponds to the *y* NullCline after the change in the value of one of the parameters of the internal elements *y*. The difference between the dotted blue line and the solid blue line accounts the important change of dynamics between the yellow trajectory (before treatment) and the green trajectory (after treatment).

In this simulation study, we proposed a toy-model (the “psychiator”) that can phenomenologically reproduce the time evolution of the intensity of psychiatric symptoms, interacting with the internal elements and his/her perceived external environmental inputs, while considering different time scales. This computational model enables to understand the effects of non-linear relations between different psychiatric disorders' determinants. It has a set of strengths and limitations that we will detail. The Figure 4 helps to show the importance of such simulations by offering a comparison of the time series of the four variables, the cyclic trajectory in the phase space of the three fast variables, and the phase plane of a subspace of the system (i.e., what “draw” the equations in space, with the visualization of the different dynamic objects of the system such as the stable or unstable fixed points).

### 4.1. Main interests

We retrieve at least five main interests of such a computational toy-model.

First, by varying the values of the parameters, such a model allows visualization of simulations of different case formulations of psychiatric conditions and disorders and aggregates of symptoms. Such visualizations allow to find potentially new endpoints for clinical and research purposes, which in themselves enables computational models to be refined. This model allows to show that the interactions between three relatively simplified variables lead to behaviors that are very difficult to intuitively interpret. This complexity thus demonstrates the need to consider non-linear relationships rather than single variable-phenotype relationships at the clinical level. For instance, for psychotherapy, such non-linear formalization of the patient behaviors can help guide indications for specific drug therapy. It can also constitute a didactic and pedagogical tool to help the patients to understand the (non-linear) factors at the origin of their distress (Burger et al., 2020; Fried and Robinaugh, 2020).

Secondly, this model provides a high flexibility, allowing a large number of concepts to be discussed and made practical. Indeed, its interest lies in the possibility of using a large number of different empirical data from research in psychiatry, clinical psychology or neuroscience, with different actions on the parameters of the toy-model, to observe in particular the inter- and intra-individual differences of psychiatric disorders. For instance, this model is sufficiently generic to be interpreted for different type of symptoms. Moreover, the versatility of this model (i.e., the model can be adapted to many different psychiatric disorders and psychological conditions) allows to compare the differential evolutions of these disorders. This comparison could help to specify their phenotypes and refine precision medicine. Psychiatry is struggling with the issues of differential diagnoses (i.e., distinguishing two disorders whose symptoms overlap) and with the issues of comorbidity (i.e., assessing the need to distinguish two conditions or to combine two of them into one). In recent decades, no diagnostic biomarker, neither predictive nor endotype have been identified to clearly define the boundaries of psychiatric disorders: in this way, hopes lies in the differential evolution of psychiatric disorders themselves, potentially evaluable with such a computational model. The very large number of possible combinations refers to the infinite number of phenotypic variations in psychiatry. Such a model provides access to the variability of psychiatric phenotypes for a same disorder.

Thirdly, on the therapeutic level, such a toy-model provides information on the attracting states (i.e., the states to which the system gravitates). This result allows to understand what stabilizes the patient in a given (healthy) state. The warning signals leading to this attracting state can thus be detected upstream (Hayes and Andrews, 2020).

Fourthly, this model proposes a dynamically theoretical framework allowing to constitute longitudinal studies and the use of assessment tools in daily life. This model provides a flexible framework allowing to integrate a large number of heterogeneous data, distinguishing patient-dependent factors, her/his subjective experience and the environment. Certainly, the absence of large cohorts of longitudinal data in psychiatry is due to numerous economic or organizational factors. However, they also relate to a lack of methodological tools. In other words, such a framework constitutes a prerequisite for the collection of longitudinal data in psychiatry. Such methods may be integrated into moment-to-moment ecological macro- or micro-level assessment (depending on the period), and especially a widely used methods such as ecological momentary assessment or joint modeling of time-to-event outcome with time-dependent predictors (i.e., given the temporal nature necessary to predict the onset of a disorder) (McGorry et al., 2014). In turn, data offered by such techniques would allow to confirm and validate this model in terms of predictivity. Regarding the clinical utility, based on these repeated evaluations in ecological daily life, individual predictions allow a patient to be informed of her/his level of risk and of the (natural or under treatment) course of her/his psychiatric disorder.

Fifthly, such a model could refine at least two kinds of nosological psychiatric models: staging models (McGorry et al., 2014) and symptom network models of psychopathology (Borsboom, 2017). One of the criticisms of these proposals is that stable and static clinical pictures at any given time could not be predicted on the basis of a sampling of cross-sectional data (McGorry et al., 2014). Cross-sectional data at a single point in time cannot provide predictions about the future emergence of a psychiatric disorder. Having multiple sets of cross-sectional data requires dynamic models to deal with the nonlinearity (i.e., the absence of the direct association between variables) (McGorry and van Os, 2013; van Os, 2013). Sets of snapshots of clinical states can be integrated in our model to provide information on the dynamic course of psychiatric disorders, in a non-linear manner. For example, symptoms networks could be modeled based on our computational dynamical model. In symptom network models, a psychiatric disorder is defined as the steady frozen state of a strongly connected network. A dynamic component could be added to this definition, especially by providing a notion of threshold corresponding to a bifurcation of the model. The evolutions and interactions between heterogeneous variables (objective, subjective or environmental) can be considered in dynamical symptom network models (e.g., based on multi-level vector autoregression models using time-series data) (Haslbeck et al., 2021).

Finally, such a ubiquitous and abstract toy-model, in future works, would allow to propose new classifications of psychiatric disorders according to their dynamics. We would find some disorders particularly sensitive to the environment (e.g., OCD), others presenting a rapid rhythmic activity (e.g., rapid cycles in bipolarity), or others with abrupt bifurcations in their trajectory.

### 4.2. Limitations

This toy-model also has several limitations.

First, the explanatory scope of this model remains limited. There could have been an infinity of models, impeding this model from being considered as predictive. Comparable dynamics could be found with completely different set of parameters, or even with different ordinary equations. Nowadays, the absolute values of parameters are not representative of any physically measurable elements. Indeed, it is important to note that the terms of the equation are contingent, and could have been defined differently. In other words, there could have been several ways of defining the dynamics of a psychiatric disorder, and this model is only one of the answers allowing to visualize their evolution over time. For example, mathematical solutions could be found to reduce the number of model parameters. However, we choose to keep it under this form to maintain intuitive clinical interpretability. To be predictive for a given patient, the model should incorporate her/his specific collected longitudinal values. Unlike digital twins (i.e., data-driven mathematical models of patients that allow for more precise and effective medical interventions), this toy-model is not built, at first, to be personalized. However, the objective of this study is not to select the best model (in terms of the equation structuring), but to propose a systematic formulation of an observed phenotypic behavior, based on the clinically relevant variables and parameters.

Secondly, this model seems reductionist regarding clinical practice. However, it integrates in an original way non-linear relations between qualitatively and clinically interpretable equations. Indeed, we have proposed a model in which it is not the biological mechanistic structures that are modeled, but behaviors (Marr, 2010). Moreover, we do not aim to directly capture internal biological elements, but rather the resulting output interacting with the symptoms and perceived environmental variables, at a very coarse level (Sulis, 2021).

Thirdly, it turns out that this model should be tested with experimental data to ensure its discriminative, construct and/or predictive validity. We hypothesize that research in psychiatry waited for such a robust model to collect empirical data, and conversely that no robust model could be built due to a lack of empirical data. The absence of measurements of such values is largely due to the absence of a model as we propose it. We are thus seeking to break this vicious circle with such a toy-model. After empirical validation, the structure of this computational model could serve as an optimized framework for simulating behavior and predicting the course of disorders. In order to choose whether certain other methods could allow to model psychiatric disorders in the same way, a set of models similar to this one should be constructed, with a sorting of these models by an analysis of the choice of the best model (in terms of choice of the free parameters). Future studies will aim to identify the maximum and minimum ranks of the (13) parameter values described in the Table 2.

Fourthly, the representation of *x* corresponds to the intensity of the symptoms, and that the model corresponds to an abstract representation of psychiatric disorders. It could be necessary to refine the model to have different kinds of symptoms. Indeed, in this model, only the symptom intensity is discussed, but not the nature of symptoms forming the dynamics. For instance, it is not possible to distinguish the effect of delirium vs. acoustico-verbal hallucinations in schizophrenia. The fluctuations do not allow to affirm whether these are depressive or maniac episodes. However, this computational model aims to model the characteristics of specific individuals (e.g., “an individual with an autism spectrum disorder”), and not a psychiatric category (e.g., “autism spectrum disorders”). Thus, the absence of characterization of the nature of the symptoms is of little importance, because our approach remains idiographic: for some individuals, the dynamic model will evolve toward a characteristic psychotic break, and for others, it will evolve toward a return to the previous state, according to the individual characteristics of the different variables.

Fifthly, a last limit concerns the potential difficulty to interpret the dynamics of the models. Indeed, the incorporated variables account for non-linear phenomena, which could be not intuitively explainable to a clinician. More precisely, it could be difficult to know why some stressors and triggers have or not an action on the system (e.g., inducing a dissociation), why certain nonlinear effects occur at particular times (e.g., fluctuations of affective states) or how interactions between certain symptoms occur (e.g., low mood and overeating or anorexia). Clinical inference from this kind of model should be very careful. By extension, it will be necessary to ensure that these individual-level models are not naively transferred to group-level models.

## 5. Conclusion

In the history of clinical psychology and psychiatry, predicting the occurrence of disorders and symptoms has focused on the evaluation of a spectrum of variables – ranging from genetics to the environment, including neurocognitive measurements or subjective feelings. These conditions are particularly difficult to model, and this difficulty is largely due to the lack of dynamic modeling to model them, despite a growing theoretical literature advancing such promises for at least several decades (Nelson et al., 2021). In order to shift from this research, we propose with this “psychiator” to dynamically modelize human behaviors and (subjective and biological) internal elements in a non-linear way, while maintaining clinical, phenomenological and biological plausibility useful to the clinician. Although this model is only a toy-model, it offers a conceptual basis for data acquisition, and can serve as a starting point based on dynamic systems for establishing a theoretical definition of psychiatric disorders and sustain nosology.

## Data availability statement

The original contributions presented in the study are included in the article/supplementary material, further inquiries can be directed to the corresponding author/s. An example code of an implementation of the model presented in this paper is available at https://github.com/ddadep/ModelPsy.

## Author contributions

CG and DD were the principal investigators and study supervisors and interpreted the results and wrote the manuscript. CG conceptualized and supervised the analysis. DD made the mathematical equation formalization and run the simulations. Both authors approved the final version.
